# Gender Differences in Service Use in a Sample of People with Schizophrenia and Other Psychoses

**DOI:** 10.1155/2012/365452

**Published:** 2012-04-03

**Authors:** Raquel Iniesta, Susana Ochoa, Judith Usall

**Affiliations:** Parc Sanitari Sant Joan de Déu, CIBERSAM, Dr. Antoni Pujadas 42, 08830 Sant Boi de Llobregat, Spain

## Abstract

*Objective*. The main objective is to analyze the use of mental health services in a sample of people with schizophrenia and other psychoses according to gender. *Method*. The sample of this observational and retrospective study (*n* = 7483) consisted of all the persons who visited any mental health service of the Parc Sanitari Sant Joan de Déu from 2001 to 2007 with a diagnosis of schizophrenia and other psychoses. The main measures analyzed regarding gender were the frequency of patients for each diagnosis, their risk of being admitted into hospital, and the number and length of hospitalizations for the subsample of inpatient people during the study period. *Results*. Men are more frequent in the total sample (58.1%). For diagnosis of schizoaffective or delusional disorder, women have a higher frequency than men. Women with diagnosis of schizophrenia have a lower risk of being admitted to the hospital (RR = 0.84, 95% CI (0.72, 0.97)). We found a higher risk of longer stays for men with schizophrenia of the disorganized type (RR = 0.49, 95% CI (0.30, 0.81)), undifferentiated (RR = 0.41, 95% CI (0.27, 0.61)), or delusional disorder (RR = 0.65, 95% CI (0.49, 0.87)). *Conclusion*. Gender of patients is a relevant variable in mental health service use by patients with schizophrenia and other psychoses.

## 1. Introduction

Gender differences in schizophrenia have received widespread empirical support with respect to incidence, age at onset, familial transmission, and neurobiological factors [1, 2]. However, gender differences in the use of mental health services have been less studied, and the results are controversial.

The most studied variables are number of hospitalizations and length of hospital stay. Some researchers have found a higher number of hospitalizations and length of stays in men than women [3–5]. However, Lindamer et al. [6] found that women with schizophrenia have a higher risk of being hospitalized than men.

Specifically, gender differences in the use of services with regard to the different subtypes of schizophrenia have been less explored. Beratis et al. [7] found that the frequency of men was more than three times greater than that of women in the residual and the catatonic subtypes. Tang et al. [8], using the ICD-10 classification system, found differences in the overall subtype distribution between male and female patients, with the paranoid subtype being more common in females; however, they did not explore hospitalizations regarding the subtypes of schizophrenia.

On the other hand, Mimica et al. [9] found a different hospitalization pattern between subtypes of schizophrenia; the catatonic subtype are admitted to hospital earlier than the paranoid subtype since the onset of the illness. The authors did not found gender influence between subtypes of schizophrenia and days prior to hospitalization.

Furthermore, it is necessary to explore gender differences with regard to mental health service use in other psychosis diagnoses. According to Høye et al. [10], psychiatrists tend to diagnose schizophrenia more often in men than women, so women might have been diagnosed with other psychoses. Therefore, the diagnosis of other psychoses should be studied in relation to prevalence and service use by gender.

However, gender differences studies in other psychosis disorders are scarce. McGlashan et al. [11] found that the profile of people with a schizoaffective disorder is similar to that of people with schizophrenia; however, the gender pattern of the two diagnoses is different, showing no gender differences in the schizoaffective disorders. In delusional disorders, course type and use of resources appeared to be similar in both genders as discussed by de Portugal et al. [12]. Substance-induced psychosis seems to be more prevalent in men, and in most cases it evolves into a schizophrenia disorder [13]. Therefore, it seems that there is no specific information about gender differences in the service use and outcome in all these diagnoses.

In first-episode psychosis, gender differences with regard to the number of hospitalizations are controversial. Cotton et al. [14] found lower hospital admission in women; however, when they analyzed affective and nonaffective psychosis separately, the differences were only found in the affective psychosis. Segarra et al. [15] did not find gender differences in the number of admissions to hospital in first-episode-psychosis non-substance-dependent patients. Malla et al. [16] did not find any gender differences in the relapse rates in a first-episode psychosis sample (including 81.7% schizophrenia, 7.9% delusional disorder, brief psychosis, and psychosis not otherwise specified, and 10% affective psychosis). Thus, it seems that patterns of service use in first-episode psychosis are different than in schizophrenia.

In general, most studies found that women with schizophrenia require less hospitalizations and shorter length of stay, but we do not know if there are any differences in relation to subtypes of diagnosis. Moreover, there is no evidence of gender differences in other psychosis diagnoses. Therefore, the aim of our study is to assess gender differences in the number of hospitalizations and length of hospital stays in schizophrenia, the subtypes of schizophrenia, and other psychosis diagnoses.

## 2. Materials and Methods

### 2.1. Study Design

Using clinical data stored in the computerized clinical records of the Parc Sanitari Sant Joan de Déu Mental Health Services Network (PSSJD), this 7-year observational and retrospective study aimed to describe and compare the use of the mental health services of the network by all the people on file with a diagnosis of “schizophrenia” or “other psychotic disorder” from 2001 to 2007. The institution has computerized clinical records operating since 2001 which enable to obtain data of the patients who have visited the health services. The Parc Sanitari Sant Joan de Déu Mental Health Services Network (PSSJD) is very wide and includes hospital and outpatient care in Barcelona and the metropolitan area. PSSJD centers are the reference mental health services in this area with a total coverage population of 800,000 people.

### 2.2. Measures and Outcomes

From all the cases included in the clinical records, only those cases with a diagnosis of “schizophrenia” or “other psychotic disorder” were selected for further analyses. The total sample size was 7483 patients. The diagnosis was that last determined as principal by the reference therapist according to the DSM-IV-R criteria. In this selected population, we analyzed gender, age of patients at the beginning of the followup, the time between the first visit in our institution and the last visit included in our study period, and a series of variables regarding use of services: the frequency of patients in each diagnose, the number of admissions in hospital care departments, and the total number of days of hospitalization in each department during the study period. Frequency of patients in each diagnosis and the risk of having at least an admission during the observed period were studied in the total sample. Number of admissions of more than 24 hours was analyzed for people who were in-patients at least a time during the analyzed period (*N* = 3755), and length of stay was studied in patients who stayed at hospital at least for a day (*N* = 2419). The difference between sample sizes was due to the presence of missing values in the variable that registered the count of number of days in hospital.

### 2.3. Ethical Aspects

All the patients treated at PSSJD signed a consent form stating that all the data regarding their clinical record is confidential and will be subject to the data protection law currently in force.

The information about patients was not included in the database, following the data protection law in order to ensure the anonymity and confidentiality of the data.

Moreover, the study was approved by the Ethics Committee of Hospital Sant Joan de Déu.

### 2.4. Statistical Analysis

The statistical analysis of the data aimed to describe gender differences with regard to the count of cases found for schizophrenia disorder, for each subtype (paranoid, disorganized, catatonic, undifferentiated, and residual) and for schizophreniform disorder, schizoaffective disorder, delusional disorder, other nonorganic psychoses, brief psychosis, shared psychotic disorder, psychotic disorder due to a general medical condition, substance-induced psychosis, and psychotic disorder NOS. The Chi-square test was used to analyze gender distribution. In order to study admission variables, two analyses were carried out. First, for the total sample we analyzed the risk of having at least one hospital admission during the study period according to gender and diagnoses by means of a logistic regression model, regardless of the total number of admissions. Second, gender differences were analyzed in the total number of patient admissions for both genders in the sample of patients admitted to any hospital department at least once during this period. Due to the overdispersion of data, the negative binomial regression with log link was used. To analyze the differences regarding the length of hospital stay per patient according to gender and diagnosis, this study took into account the patients admitted at least for a day in any of the hospital departments during the study period. Since the day count was also significantly overdispersed for each diagnosis, data was modeled once again according to the negative binomial regression model. All the models have been adjusted by age and years of treatment to avoid a confounding effect. Confidence intervals were calculated at 95%. Analyses were carried out with R 2.12.0.

## 3. Results

The sample analyzed in this study consisted of a total of 7483 patients, 3,137 (41.9%) females and 4,346 (58.1%) males with a diagnosis of “schizophrenia” with the subtype reported or with a diagnosis of “other psychotic disorder.” The mean age of men in this sample was 45.30 (SD 17.09) and 47.67 (SD 15.55) for women. The average number of years of treatment for men was 11 (SD 12.16) and for women 7.25 (SD 5.05). Both age and years of treatment values showed statistical differences between genders (*P* value < 0.001). As shown in Table 1, for the whole sample, diagnoses of “schizophrenia” or “other psychotic disorder”, men had a higher frequency (58.1%) than women (*P* value < 0.001). When analyzing by type, only the catatonic subtype of schizophrenia, other nonorganic psychosis, brief psychosis, shared psychotic disorder, and those diagnosed with a psychotic disorder due to a general medical condition had equal gender distribution (see Table 1). For all of the others, men had a higher frequency with the exception of schizoaffective and delusional disorder, showing statistically significant higher frequency for women. Regardless of the type of diagnosis, a total of 3719 (49.70%) patients were admitted to the hospital. The risk of being admitted for the first time was less in women for the whole sample (OR = 0.90, 95% CI (0.82, 0.99)) and for those women diagnosed with schizophrenia (OR = 0.84, 95% CI (0.72, 0.97)) (Table 2). Women diagnosed with brief psychosis also showed a lower risk of being admitted than men (OR = 0.64 95% CI (0.44, 0.94)).  Table 3 shows the number of admissions to hospital in patients who were admitted at least once during the study period by gender (*n* = 2419). In this sample, regardless subtypes of diagnosis, there was no statistical difference between women and men. Women diagnosed with catatonic schizophrenia in a sample of 10 patients showed a higher risk of a new admission than men (RR = 4.40, 95% CI (1.67, 11.62)).  Table 4 shows the number of days in hospital for patients who were admitted at least once by gender. The median of length of stay in hospital was 32.5 days with a range of 1–2586 for women and 47 days with a range of 1–2586 for men. There was a statistically lower risk for adding an additional day for women (RR = 0.78, 95% CI (0.72, 0.85)). Differentiating by diagnosis, men had a statistically significant higher risk of being admitted to hospital more days than women if they had schizophrenia of the disorganized type (RR = 0.49, 95% CI (0.30, 0.81)), undifferentiated (RR = 0.41, 95% CI (0.27, 0.61)), and delusional disorder (RR = 0.65, 95% CI (0.49, 0.87)).

## 4. Discussion

The first result of our study regards health care use prevalence, which is higher in men than in women for the total sample and for schizophrenia cases. These results concur with the findings from other studies [3] and are in line with results showing that men have a higher risk of developing schizophrenia [17]. The finding that women have a higher prevalence of service use for schizoaffective disorders concurs with the majority of studies which have found that affective disorders are more common in women [18]. Our results on delusional disorders also coincide with another finding [12], which finds the female-to-male ration to be 1.6 : 1.

Our results on the risk of hospitalization show that women are admitted to the hospital less than men for the general sample, the schizophrenia patient sample, and the paranoid subtype, which is the most common. These results concur with the findings of our team in a previous study. In a follow-up study at 2 years for a sample of 200 patients with schizophrenia, we found that men had a higher number of hospitalizations [19]. This also coincides with Uggerby et al. [4], who studied the prevalence of institutionalized and noninstitutionalized patients with schizophrenia in Denmark in a sample of 22,395 people. The results showed that being male was one of the predictors of institutionalization. Similarly, in a revision of 388 hospital records, Agbir et al. [5] found that men were more frequently admitted than women. However, Lindamer et al. [6] found that women with schizophrenia have a higher risk of being hospitalized than men.

As regards other diagnoses, no differences have been found with regard to risk of hospitalization, except in brief psychoses in which women also have a lower risk of hospitalization than men. These data do not coincide with previous studies in first psychotic episodes which found no difference in the pattern of service use with regard to gender [14, 15]. However, it is important to note that brief psychoses comprise a small percentage of people with first psychotic episode.

We also specifically studied the influence of gender in the subgroup of patients who have been hospitalized at least once, and we found differences in the total sample but not in schizophrenia patients. This result concurs with the findings made by our team in a study which has been mentioned above [19] and could explain why our hospitalized female patients may be more severe than males.

In reference to the results on length of hospital stay, the results for the general sample show that men have a longer stay. In our study in 2003 [19], we also found this difference. However, the previously mentioned study by [5] found that length of stay was similar in men and women.

In conclusion, our data indicate that gender affects service use in patients with psychotic disorders and that, in general, women have a better disease course than men with regard to number of hospitalizations and days of hospitalization; however, gender does not have the same impact on all schizophrenia subtypes or different psychotic disorders.

Some strengths of our study are that the data were collected from clinical records in a computerized registry and we have analyzed the total treated population over 7 years in a large section of the Barcelona metropolitan area which represents 800,000 people. The results from this study give us an overview of the type of care received by men and women with psychotic disorders who are treated in mental health.

### 4.1. Limitations

Our data only account for public mental health services, and it referred to the information registered by our institution. We do not have previous clinical information for patients included in this study, although it should be emphasized that the public national health system is the most frequently used health service in Spain, especially for people who suffer from severe mental disorders. Furthermore, most of the centers of reference of our area are depressed areas that use more public services.

Admission rates do not directly represent clinical need or morbidity differences, but rather only the use of existing mental health services.

Given that our data have been extracted solely from clinical records, many of the diagnoses are not made by means of a structured clinical interview. For people with more than one diagnosis, we have included the principal diagnosis determined by the reference therapist in the last visit included in the study, according to DSM-IV-R criteria.

In some of the diagnoses, the samples are small and might prevent differences from being found, or the differences found are not clinically significant.

### 4.2. Practical Implications

The main result of the study is the relevance of the gender variable in the use of mental health services by patients with schizophrenia and other psychoses. Perhaps this variable must be taken into account in future studies as needs assessments and use of mental health resources in these patients populations [20].

## Figures and Tables

**Table 1:** Frequence of attended diagnosis by gender.

	Female	Male	Total	*P* value^†^
Schizophrenia *(whole sample) *	1140 (31.76)	2449 (68.24)	3589	<0.001
Subtypes				
Paranoid	695 (32.1)	1469 (67.9)	2164	<0.001
Disorganized	51 (30.2)	118 (69.8)	169	<0.001
Catatonic	8 (53.3)	7 (46.7)	15	0.9
Undifferentiated	108 (35.9)	193 (64.1)	301	<0.001
Residual	278 (29.6)	662 (70.4)	940	<0.001
Schizophreniform disorder	86 (37.1)	146 (62.9)	232	<0.001
Schizoaffective disorder	438 (59.7)	296 (40.3)	734	<0.001
Delusional disorder	619 (59.1)	428 (40.9)	1047	<0.001
Other psychoses nonorganic	53 (48.6)	56 (51.4)	109	0.848
Brief psychosis	115 (51.8)	107 (48.2)	222	0.6385
Shared psychotic disorder	10 (76.9)	3 (23.1)	13	0.0961
Psychotic disorder medical cond.	15 (48.4)	16 (51.6)	31	0.9
Substance-induced psychosis	2 (4.2)	45 (95.8)	47	<0.001
Psychotic disorder NOS	659 (45.2)	800 (54.8)	1459	<0.001

Total *n* = 7483	3137 (41.9)	4346 (58.1)	7483	<0.001

*n* (%) ^†^ *χ*² Chi-square test.

**Table 2:** Adjusted odds ratio of being admitted at least a time to the hospital by gender.

	OR^†^	CI 95%
Schizophrenia *(whole sample) *	0.84	(0.72, 0.97)**
Subtypes		
Paranoid	0.83	(0.68, 1.01)*
Disorganized	1.07	(0.53, 2.17)
Catatonic	1.34	(0.01, 171.72)
Undifferentiated	0.96	(0.59, 1.57)
Residual	0.75	(0.55, 1.02)*
Schizophreniform disorder	1.11	(0.64, 1.92)
Schizoaffective disorder	0.91	(0.67, 1.22)
Delusional disorder	1.08	(0.83, 1.41)
Other psychoses nonorganic	0.77	(0.36, 1.65)
Brief psychosis	0.64	(0.44, 0.94)**
Shared psychotic disorder	9.28	(0.12, 744.57)
Psychotic disorder medical cond.	5.78	(0.42, 78.83)
Substance-induced psychosis	0.85	(0.05, 14.95)
Psychotic disorder NOS	1.19	(0.96, 1.48)

Total *n* = 7483	0.90	(0.82, 0.99)**

^†^Odds ratio of being admitted to the hospital at least once by gender, adjusted by age and years of treatment. Men are the reference category.

*P value < 0.1.

**P value < 0.05.

**Table 3:** Number of admissions in hospital in patients who have been admitted at least one time by gender.

	*n* ^†^		Nadmission^‡^			
	Female	Male	Female	Male	RR^1^	CI 95%
Schizophrenia *(whole sample) *	588	1508	2 (1–43)	2 (1–49)	1.08	(0.96, 1.21)
Subtypes						
Paranoid	368	888	1.5 (1–23)	2 (1–25)	0.98	(0.85, 1.14)
Disorganized	33	80	2 (1–25)	2 (1–20)	1.27	(0.79, 2.03)
Catatonic	5	5	5 (1–8)	2 (1–4)	4.40	(1.67, 11.62)**
Undifferentiated	54	100	2.5 (1–37)	2 (1–27)	1.40	(0.96, 2.04)*
Residual	128	435	2 (1–43)	1 (1–49)	1.08	(0.86, 1.36)
Schizophreniform disorder	41	67	1 (1–5)	1 (1–14)	1.39	(0.93, 2.08)
Schizoaffective disorder	233	170	2 (1–37)	2 (1–26)	1.11	(0.88, 1.39)
Delusional disorder	220	138	1 (1–15)	1 (1–20)	1.03	(0.79, 1.34)
Other psychoses nonorganic	23	26	1 (1–5)	1 (1–5)	1.58	(0.96, 2.58)
Brief psychosis	45	54	1 (1–3)	1 (1–7)	1.24	(0.84, 1.84)
Shared psychotic disorder	4	0	*na*	*na*	*na*	*na*
Psychotic Disorder medical cond.	3	3	*na*	1 (1–20)	*na*	*na*
Substance-induced psychosis	1	22	*na*	1 (1–4)	1.00	(0.06, 15.99)
Psychotic disorder NOS	283	326	1 (1–16)	1 (1–24)	0.99	(0.80, 1.22)

Total *n* = 3755	1441	2314	1 (1–43)	1 (1–49)	1.02	(1.01, 1.03)*

^†^Number of persons who have been admitted at least one time in hospital.

^‡^Median and range of admissions during the study period.

^1^RR of adding a new admission for patients who have been admitted at least once, regarding gender, adjusted by age and years of treatment.

Men are the reference category.

*P value < 0.1.

**P value < 0.05.

**Table 4:** Number of days in hospital in patients who have been admitted at least one time by gender.

	*n* ^†^		*n* days^‡^			
	Female	Male	Female	Male	RR^1^	CI 95%
Schizophrenia *(whole sample) *	351	949	64 (1–2586)	92 (1–2586)	0.92	(0.81, 1.05)
Subtypes						
Paranoid	217	579	51 (1–2586)	56 (1–2586)	0.99	(0.84, 1.16)
Disorganized	24	62	134 (24–1142)	253 (1–2586)	0.49	(0.30, 0.81)**
Catatonic	4	3	1887 (664–2586)	2586 (1960–2586)	1.04	(0.00, 486.6)
Undifferentiated	40	67	37 (6–2586)	105 (1–2586)	0.41	(0.27, 0.61)***
Residual	66	238	70.5 (6–2586)	1165 (1–2586)	0.76	(0.57, 1.02)*
Schizophreniform disorder	27	37	22 (1–103)	27 (4–141)	1.04	(0.60, 1.79)
Schizoaffective disorder	153	106	49 (1–2586)	58.5 (1–2586)	1.23	(0.95, 1.59)
Delusional disorder	139	78	30 (1–2293)	39.5 (12586)	0.65	(0.49, 0.87)**
Other psychoses nonorganic	4	6	32 (9–2586)	14 (6–21)	1.17	(0.17, 8.20)
Brief psychosis	39	48	10 (1–84)	12 (1–106)	1.05	(0.68, 1.62)
Shared psychotic disorder	3	0	19 (7–22)	*na*	*na*	*na*
Psychotic Disorder medical cond.	1	1	*na*	*na*	*na*	*na*
Substance-induced psychosis	0	8	*na*	57 (2–1615)	1.02	(0.93, 1.11)
Psychotic disorder NOS	207	262	18 (1–2586)	17 (1–2586)	0.85	(0.70, 1.04)

Total *n* = 2419	924	1495	32.5 (1–2586)	47 (1–2586)	0.78	(0.72, 0.85)***

^†^Number of persons who have been admitted at least one day in hospital.

^‡^Median and range of days during the study period.

^1^RR of adding a new day of hospitalisation for patients who have been admitted at least once, regarding gender, adjusted by age and years of treatment.

Men are the reference category.

*P value < 0.1.

**P value < 0.05,

***P value < 0.001.
